# Supplementary material for: Maintenance of Type 2 Response by CXCR6-Deficient ILC2 in Papain-Induced Lung Inflammation
Source: Int J Mol Sci. 2019 Nov 4;20(21):5493. doi: 10.3390/ijms20215493 (PMC6862482; doi:10.3390/ijms20215493)
Supplement: Supplementary file 1 [file ijms-20-05493-s001.pdf]

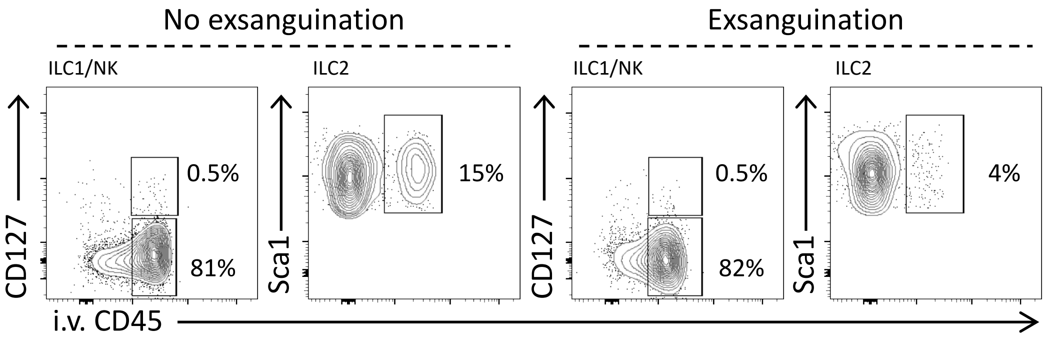

Figure S1. Exsanguination is efficient to remove circulating ILC2. Flow cytometry analyses of ILC1/NK (Lin$^-$(CD3, CD5, CD19, TCRβ, TCRγδ, Gr1, Ter119, CD8α, F4/80) NK1.1$^+$ NKp46$^+$) and ILC2 (Lin$^-$(CD3, CD5, CD19, TCRβ, TCRγδ, Gr1, Ter119, CD8α, F4/80) NK1.1$^-$ IL7R$^+$ Sca1$^+$) lung cells from CXCR6$^{+/GFP}$ and CXCR6$^{GFP/GFP}$ mice. Different subsets are distinguished by the expression of CD45. Anti-CD45 antibody coupled with a fluorochrome was injected intravitally 2 minutes before the sacrifice.

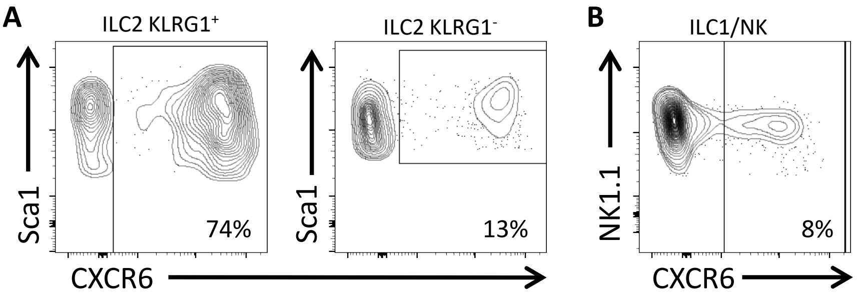

Figure S2. CXCR6 is mainly expressed by lung ILC2 KLRG1+. (A) Flow cytometry analyses of $CD45^+$ Lin^- (CD3, CD5, CD19, TCRβ, TCRγδ, Gr1, Ter119, CD8α and F4/80) $NKp46^-$ $NK1.1^-$ lung cells from $CXCR6^{+/GFP}$ mice. Different subsets are distinguished depending on the expression of KLRG1. Sca1 and CXCR6 expression from $KLRG1^+$ and $KLRG1^-$ populations (graphs are representative of n=3). (B) Flow cytometry analyses of $CD45^+$ Lin^- lung cells from $CXCR6^{+/GFP}$ mice. CXCR6 expression among ILC1/NK ($NK1.1^+$ $NKp46^+$) cells (graphs are representative of n=3).

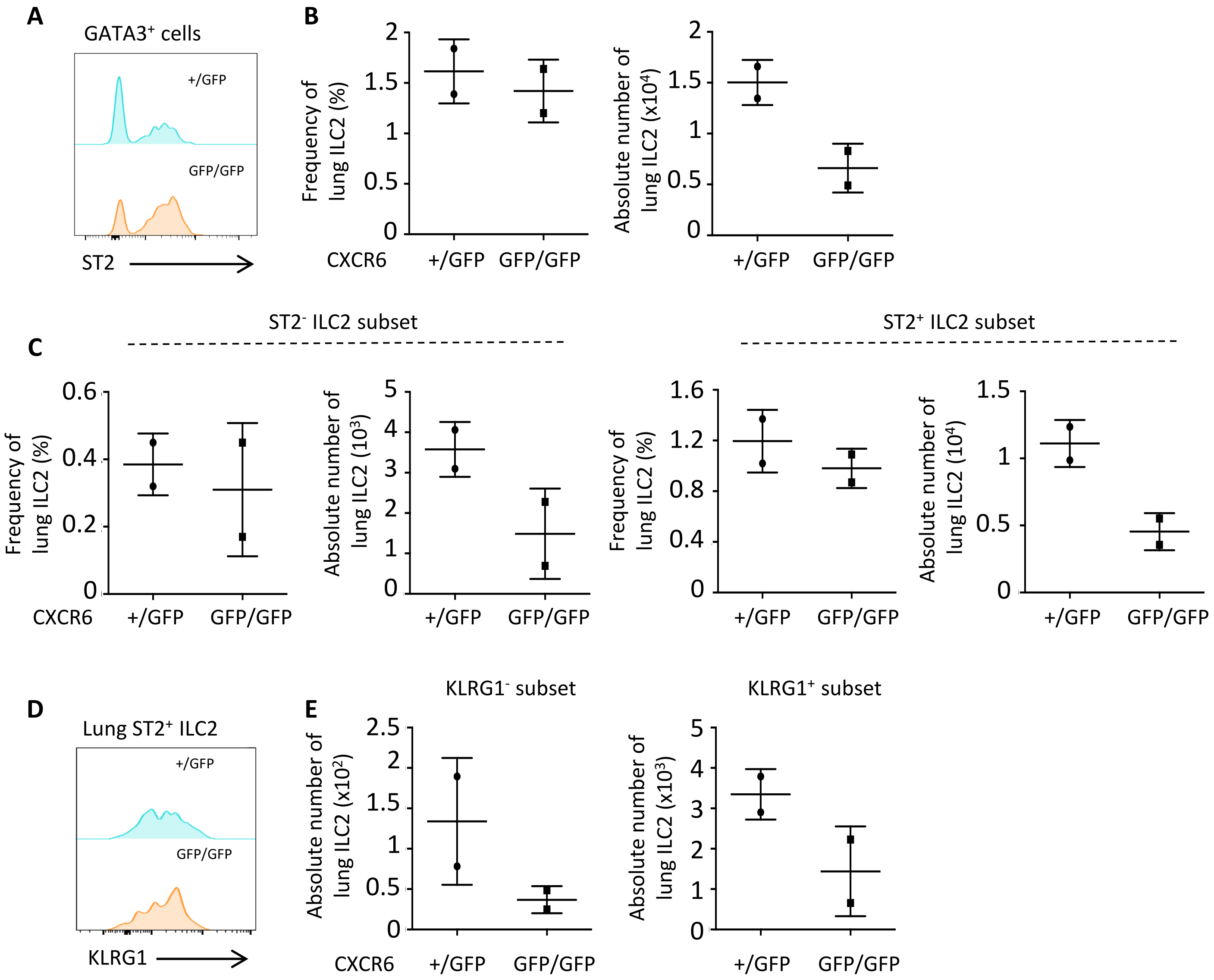

Figure S3. Phenotype and frequency of ILC2 are altered in CXCR6$^{GFP/GFP}$ mice after inflammatory induction (papain stimulation) (A) ST2 MFI of GATA3^{+} lung cells from CXCR6$^{+/GFP}$ and CXCR6$^{GFP/GFP}$ mice. (+/GFP = 2, GFP/GFP = 2) (B) Frequency and absolute number of lung ILC2 (CD45^{+} Lin^{-} GATA3^{+}) from CXCR6$^{+/GFP}$ and CXCR6$^{GFP/GFP}$ mice. (+/GFP = 2, GFP/GFP = 2) (C) Frequency and absolute number of lung ST2^{-} ILC2 and ST2^{+} ILC2 (CD45^{+} Lin^{-} GATA3^{+}) from CXCR6$^{+/GFP}$ and CXCR6$^{GFP/GFP}$ mice. (+/GFP = 2, GFP/GFP = 2) (D) KLRG1 MFI of GATA3^{+} ST2^{+} lung cells from CXCR6$^{+/GFP}$ and CXCR6$^{GFP/GFP}$ mice. (+/GFP = 2, GFP/GFP = 2) (E) Frequency and absolute number of KLRG1^{+} ST2^{+} and KLRG1^{-} ST2^{+} lung ILC2 (CD45^{+} Lin^{-} GATA3^{+}) from CXCR6$^{+/GFP}$ and CXCR6$^{GFP/GFP}$ mice. (+/GFP = 2, GFP/GFP = 2).

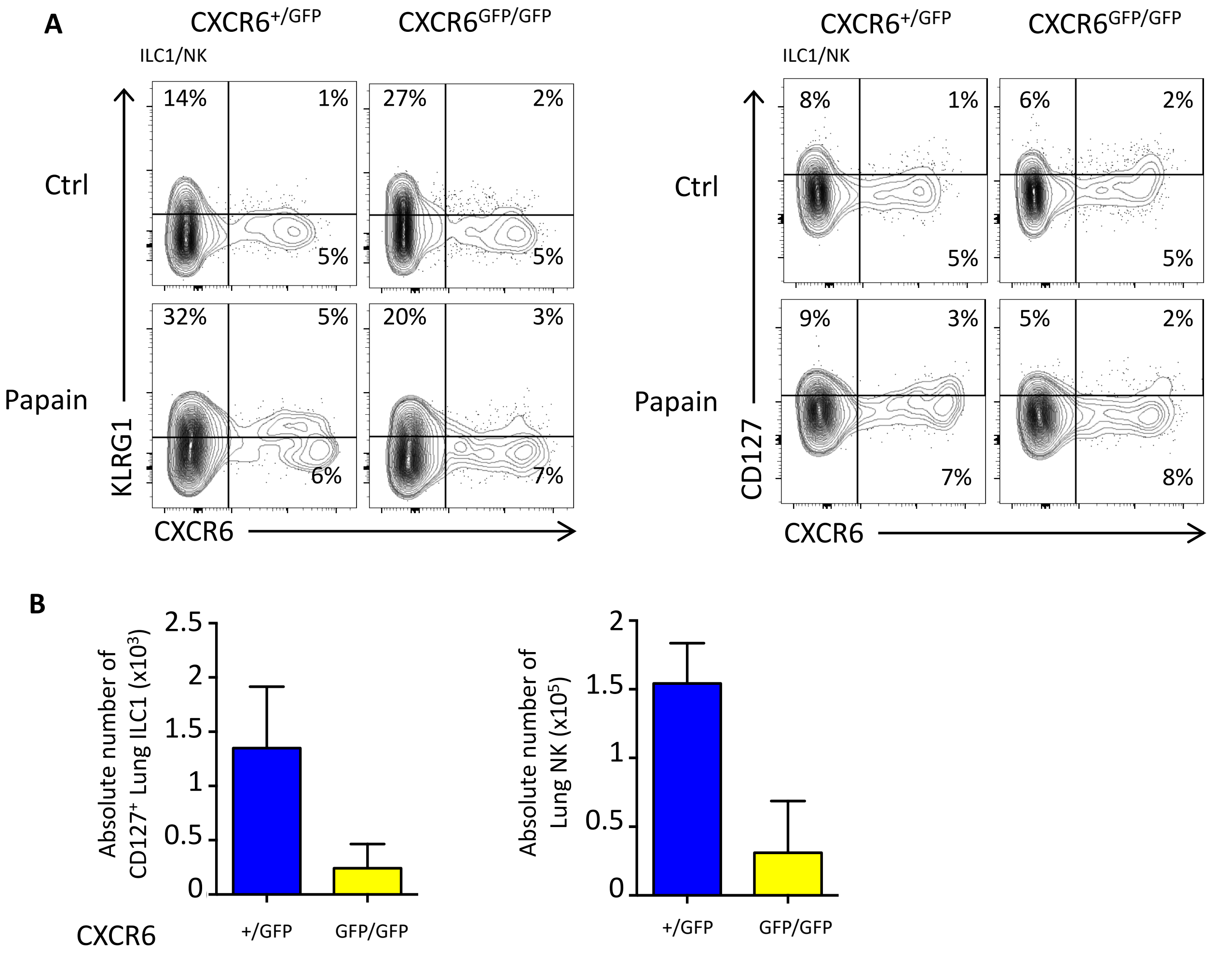

Figure S4. ILC1 and NK cell numbers are altered in CXCR6$^{GFP/GFP}$ mice after inflammatory induction (papain stimulation). (A) Flow cytometry analyses of ILC1/NK (Lin^{-}:CD3, CD5, CD19, TCRβ, TCRγδ, Gr1, Ter119, CD8α, F4/80; CD45^{+} NK1.1^{+} NKp46^{+}) lung cells from CXCR6$^{+/GFP}$ and CXCR6$^{GFP/GFP}$ mice. Different subsets are distinguished by the expression of KLRG1 and CXCR6 (left panel of graphs) or CD127 and CXCR6 (right panel of graphs). (+/GFP = 2, GFP/GFP = 2). (B) Absolute numbers of ILC1 (lin^{-} CD45^{+} NKp46^{+} NK1.1^{+} CD127^{-}) and NK cells (Lin^{-} CD45^{+} NKp46^{+} NK1.1^{+} CD127^{+}) from CXCR6$^{+/GFP}$ and CXCR6$^{GFP/GFP}$ mice. (+/GFP = 2, GFP/GFP = 2).

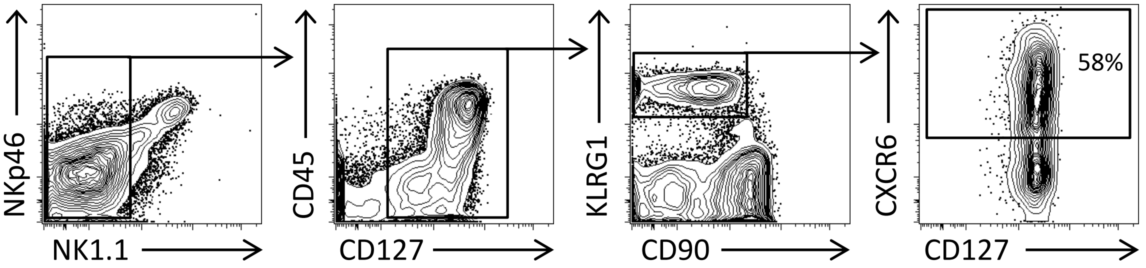

Figure S5. CXCR6 is expressed by *lamina propria* (LP) ILC2. Flow cytometry analyses of LP ILC2 (Lin$^-$:CD3, CD5, CD19, TCRβ, TCRγδ, Gr1, Ter119, CD8α and F4/80; NKp46$^-$ NK1.1$^-$ CD127$^+$ KLRG1$^+$) from CXCR6$^{+/GFP}$ mice. CD127 and CXCR6 expression among ILC2 cells (graphs are representative of n=5).
